# Measuring and decomposing inequity in self-reported morbidity and self-assessed health in Thailand

**DOI:** 10.1186/1475-9276-6-23

**Published:** 2007-12-18

**Authors:** Vasoontara Yiengprugsawan, Lynette LY Lim, Gordon A Carmichael, Alexandra Sidorenko, Adrian C Sleigh

**Affiliations:** 1 National Centre for Epidemiology and Population Health (NCEPH), ANU College of Medicine and Health Sciences, Australian National University, Canberra ACT 0200, Australia; 2 Australian Centre for Economic Research on Health (ACERH), ANU College of Medicine and Health Sciences, Australian National University, Canberra ACT 0200, Australia

## Abstract

**Background:**

In recent years, interest in the study of inequalities in health has not stopped at quantifying their magnitude; explaining the sources of inequalities has also become of great importance. This paper measures socioeconomic inequalities in self-reported morbidity and self-assessed health in Thailand, and the contributions of different population subgroups to those inequalities.

**Methods:**

The Health and Welfare Survey 2003 conducted by the Thai National Statistical Office with 37,202 adult respondents is used for the analysis. The health outcomes of interest derive from three self-reported morbidity and two self-assessed health questions. Socioeconomic status is measured by adult-equivalent monthly income per household member. The concentration index (CI) of ill health is used as a measure of socioeconomic health inequalities, and is subsequently decomposed into contributing factors.

**Results:**

The CIs reveal inequality gradients disadvantageous to the poor for both self-reported morbidity and self-assessed health in Thailand. The magnitudes of these inequalities were higher for the self-assessed health outcomes than for the self-reported morbidity outcomes. Age and sex played significant roles in accounting for the inequality in reported chronic illness (33.7 percent of the total inequality observed), hospital admission (27.8 percent), and self-assessed deterioration of health compared to a year ago (31.9 percent). The effect of being female and aged 60 years or older was by far the strongest demographic determinant of inequality across all five types of health outcome. Having a low socioeconomic status as measured by income quintile, education and work status were the main contributors disadvantaging the poor in self-rated health compared to a year ago (47.1 percent) and self-assessed health compared to peers (47.4 percent). Residence in the rural Northeast and rural North were the main regional contributors to inequality in self-reported recent and chronic illness, while residence in the rural Northeast was the major contributor to the tendency of the poor to report lower levels of self-assessed health compared to peers.

**Conclusion:**

The findings confirm that substantial socioeconomic inequalities in health as measured by self-reported morbidity and self-assessed health exist in Thailand. Decomposition analysis shows that inequalities in health status are associated with particular demographic, socioeconomic and geographic population subgroups. Vulnerable subgroups which are prone to both ill health and relative poverty warrant targeted policy attention.

## Background

Ever since public health leaders, at least 150 years ago, began using systematic information on population subgroups there has been concern about adverse health effects of inequitable development [1-3]. Over the past few decades, studies have measured socioeconomic inequalities and have linked these to inequalities in population health [4-6]. Recent evidence worldwide has consistently shown that morbidity and mortality are concentrated at the lower end of the socioeconomic spectrum [7-9]. In developing countries, gaps in health-related outcomes between the rich and the poor can be large [10-13]. These limit poor peoples' potential to contribute to the economy by reducing their capacity to function and live life to the fullest. But besides having instrumental value, health also has intrinsic value [14], so that inequalities in health directly affect the well-being and happiness of the poor [15]. The study of poor-rich inequalities in health status should not, however, solely quantify their magnitude. Research should also identify which population subgroups are the most disadvantaged. Once this is known it becomes possible to identify the determinants of inequalities, including those associated with age, gender, education, occupation and geographical location. These variables have previously been identified as powerful sources of health inequalities in low and middle income countries [14,16,17].

To date, study of health inequalities in developing countries has tended to focus on questions of equity in health care and health care delivery rather than on the distribution of health across social and economic subgroups of the population. This study adopts the International Society for Equity in Health (ISEqH) framework, which defines equity in health as "the absence of potentially remediable, systematic differences in one or more aspects of health across socially, economically, demographically, or geographically defined populations or subgroups" [18]. Understanding the magnitude and determinants of inequities in health is vital to generating essential information for policy decisions, and has obvious implications for targeting vulnerable groups.

This paper reports ongoing research on the problem of health inequity in Thailand, a developing economy that has grown steadily for 50 years and is now approaching middle-income status with an average annual per capita income in 2005 of $US2750 [19]. Like many developing countries, Thailand has faced problems of poverty and economic inequalities. Its new official poverty line in 2002 revealed that 17.7 percent of the population in the Northeast were living below the national poverty line, compared to 9.8 percent in the North, 8.7 percent in the South, 4.3 percent in the Central region outside Bangkok, and 0.5 percent in Bangkok [20]. These geographical differences have translated into differences in health-related outcomes [21]. The *Thailand Health Profile 1999–2000 *and *2001–2004 *[22,23] issued by the Thai Ministry of Public Health reveals official concern over the unequal allocation of health resources and regional variation in health outcomes, with the highest life expectancy in Bangkok (83 for women and 75 for men) and the lowest in the North (73 for women and 67 for men). In addition, infant mortality in rural areas is 1.85 times what it is in urban areas [24]. Also, a recent multi-level analysis study found socioeconomic inequalities in adult mortality in Thailand at both provincial and district levels [25].

Thailand has attempted to address the concern over inequalities in health-related outcomes, and in particular those in the use of health services, by introducing in 2001 a Universal Coverage Scheme. This was prompted by section 52 of the 1997 Constitution which states that "All Thai people have an equal right to access quality health services", and aimed to provide Thais with health services that were both accessible and equitable. Since then, monitoring the impacts of this scheme on health status, use of health services and healthcare expenditure has been the major challenge in advancing health system equity.

In developed countries, self-reported health is widely used to reflect individual perceptions of health and is related to socioeconomic status [26,27]. There is a good basis for using self-rated health as an outcome. It can provide a more holistic view of health which may not be reflected in objective measures such as those based on specific medical diagnoses [28,29]. In developing countries by contrast, the few initial studies of health inequalities, guided by the data available, have measured mainly inequalities in child mortality and malnutrition in children [30-32]. As many such countries are now more focused on health outcomes as part of their development strategies, and as they are moving through demographic and epidemiological transitions, survey data on self-reported morbidity and self-assessed health are becoming available. But only a limited number of studies have to date analysed such data, for countries such as China, India and Indonesia [33,34], and none of these studies have used decomposition analysis.

The objectives of this paper are two-fold: first, to use a concentration index (CI) to quantify the socioeconomic distribution of self-reported morbidity and self-assessed health in Thailand; and second, to 'decompose' these inequalities by quantifying the contributions attributable to age, sex, household type, income group, education, work status and geographic location. Decomposition analysis of self-assessed health data has been mainly undertaken for OECD and other developed countries; its application to developing countries is to our knowledge novel [30,35,36]. Previously, decomposition analyses of health inequalities in developing countries have used more objective health outcome measures; for example, a study of malnutrition inequalities in Vietnam [37] and recently one of socioeconomic inequalities in infant mortality in Iran [38]. This paper decomposes not only self-reported morbidity but also self-assessed health in Thailand for the first time using data from the Thai Health and Welfare Survey 2003. It thus seeks to identify the population subgroups most affected by health inequity in Thailand so that they can be targeted by future health development policies and strategies.

## Methods

### Source of data, health outcome variables and their determinants

Household surveys are common sources of data in developing countries [39]. Of particular interest to this study are the Thai Health and Welfare Surveys (HWSs) conducted for the first time in 1974 then again in 1976, and at five-year intervals thereafter until, after implementation of the Universal Coverage Scheme in 2001, surveys were conducted annually until 2005 to monitor its impact. In these surveys every member of a participating sample household aged 15 years or older is interviewed about their morbidity (including injuries and disabilities), health-seeking behaviour and illness expenditure. Data used here are from the 2003 HWS, which covers 68,433 individuals from 19,952 households. Children aged less than 15 years, 23 percent of the total sample, were excluded; 37,202 (72 percent) of the remainder responded to both the self-reported morbidity and self-assessed health questions and were included in the analysis. The number of men and women absent from the household at the time of survey was 8,182 (34.1 percent of eligible males) and 6,646 (23.7 percent of eligible females). Proxy responses were elicited for basic household socioeconomic and demographic questions (e.g. household income), but not for individual health questions (e.g. self-reported health). Data were weighted to represent the structure of the Thai population using weighting factors provided with the HWS. All statistical analyses were performed using STATA version 9 [40].

Table 1 presents means and concentration indices for five health outcomes (three self-reported morbidity measures and two self-assessed health measures) and a series of potential determinants of those outcomes. The outcome measures are:

**Table 1:** Mean and Concentration Indices of health outcome variables and their determinants for 37,202 respondents

**DEPENDENT/HEALTH OUTCOME VARIABLES**(yes = 1, otherwise = 0)	**Proportion**	**Concentration Index**
Recently ill ('ill or not feeling well' in last month)	0.224	-0.099
Chronic illness (lasting more than 3 months) during past month	0.225	-0.085
Hospital admission (during the past 12 months, excluding maternity admissions)	0.059	-0.103
Health compared to a year ago worse or much worse	0.199	-0.139
Health compared to peers (same age, sex, socioeconomic status and lifestyle) worse or much worse	0.131	-0.174

**INDEPENDENT VARIABLES/DETERMINANTS **(yes = 1, otherwise = 0)	**Proportion**	**Concentration Index**
***Demographic characteristics***		
Males aged 15–29	0.143	0.050
Males aged 30–44	0.144	0.085
Males aged 45–59	0.103	0.045
Males aged 60+	0.061	-0.197
Females aged 15–29	0.170	0.032
Females aged 30–44	0.176	0.054
Females aged 45–59	0.123	-0.008
Females aged 60+	0.079	-0.186
**Sub-total**	1.000	
One-person male household	0.023	0.156
One-person female household	0.022	-0.104
Household with no dependent	0.279	0.197
Household with dependent children but no elderly	0.419	-0.025
Household with elderly	0.209	-0.121
Household with only dependents, no working-age members	0.047	-0.259
**Sub-total**	1.000	
***Socioeconomic characteristics***		
Income quintile: 1 – lowest 20%	0.236	-0.848
Income quintile 2 – lower 20%	0.211	-0.365
Income quintile 3 – middle 20%	0.181	0.037
Income quintile 4 – higher 20%	0.188	0.420
Income quintile 5 – highest 20%	0.183	0.767
**Sub-total**	1.000	
Education: primary level	0.641	-0.141
Education: secondary level	0.258	0.134
Education: higher level	0.101	0.466
**Sub-total**	1.000	
Work status: agriculture and fishery	0.295	-0.424
Work status: elementary occupation	0.090	0.057
Work status: others including professionals, technicians, or service workers	0.355	0.318
Not in workforce: housewife	0.081	-0.049
Not in workforce: disabled	0.015	-0.320
Not in workforce: others such as decided not to work or student	0.164	-0.169
**Sub-total**	1.000	
***Geographic characteristics (resident in)***		
Bangkok	0.139	0.508
Urban Central excluding Bangkok	0.073	0.148
Rural Central	0.142	0.097
Urban North	0.041	0.031
Rural North	0.154	-0.380
Urban Northeast	0.055	0.028
Rural Northeast	0.286	-0.825
Urban South	0.024	0.068
Rural South	0.086	-0.043
**Sub-total**	1.000	

1) Whether 'ill or not feeling well' during the past month (i.e., whether 'recently ill');

2) Whether suffered from a chronic illness (one that had lasted for more than 3 months) during the past month;

3) Whether admitted to a hospital during the past 12 months (excluding maternity admissions);

4) Self-assessed health to be worse or much worse compared to a year ago;

5) Self-assessed health to be worse or much worse compared to others of the same age, sex, socioeconomic status and lifestyle (i.e., compared to 'peers').

The most common recent illnesses reported were diseases of the respiratory system (27.8 percent), and the most common conditions identified by the chronically ill were cardiovascular diseases (33.1 percent). Hospitalizations of females for maternity purposes were excluded from the analysis because these were not considered 'illness' conditions. The most commonly reported reasons for non-maternity hospital admissions were diseases of the digestive system (18.5 percent).

The two self-assessed health variables focus on perceptions of recent deterioration in one's health and perceptions that one is less healthy than is normal in one's peer group. These health outcomes are examined because they are the only self-rated outcomes for which data are available in the 2003 HWS, but they do tap psychologically significant dimensions of health – the notion that one's health is in decline, and that one is less healthy than might be hoped given one's demographic and social circumstances.

Determinants considered in seeking to account for observed probabilities of reporting the three types of morbidity and the two measures of self-rated health were chosen having regard to (i) the sorts of determinants examined in previous similar studies for developed countries and (ii) what was available in the dataset being used. They were:

1) ***demographic characteristics***, which consisted of eight age-sex interaction categories combining males and females with four age groups (15–29, 30–44, 45–59 and 60 years or older), and six household type categories (one-person male, one-person female, multi-person households with at least one working age member and no dependents, dependent children only and dependent elderly, and multi-person households with no working age member). Males aged 15–29 years and 'household with no dependent' were treated as reference groups, and proportions in various categories were as indicated in the 'Proportion' column of Table 1.

2) ***socioeconomic characteristics***, which comprised income, education and economic activity. Adult-equivalent household income per household member, the derivation of which is described below, was grouped into five quintiles; three levels of education ('primary', 'secondary' and 'higher') were adopted, with 'higher' (more than 12 years) as the reference group; and economic activity consisted of three occupational and three 'Not in workforce' categories. Two occupation categories were 'Agriculture and fishery' and 'Elementary occupation' (including the likes of street vendors, domestics, and non-agricultural labourers), with the third, 'Others, including professionals, technicians and service workers' treated as the reference group. The three 'Not in workforce' groups were housewives, the disabled and 'Others'. The occupation groups selected were designed to isolate those in lower status occupations from other employed respondents, while the three 'Not in workforce groups' sought to separate those who were in that situation for maternal/domestic, medical and other (e.g., unemployment, educational) reasons.

3) ***geographic characteristics***, which consisted of eight urban-rural and region (Northeast, North, Central, South) interaction categories plus Bangkok, with 'urban Central (excluding Bangkok)' as the reference group. The four regions of Thailand at the 2000 Census accounted for 34.2 percent (Northeast), 18.8 percent (North), 23.3 percent (Central) and 13.3 percent (South) of the national population of 60.9 million, Bangkok accounting for the other 10.4 percent. Regional proportions of population rural were, respectively, 83.3 percent, 79.3 percent, 65.5 percent and 77.0 percent. Regional poverty levels were given earlier. They show poverty in the Northeast to be almost double the level in the next poorest region (the North), and four times the level in the Central region (outside Bangkok).

### Measurement of socioeconomic status

Household monthly per capita income is used as the socioeconomic measure. An attempt was made to interview every adult member of a household, but as already indicated, in the case of continued absence after three visits another household member could respond on behalf of an absent member (except to self-reported morbidity and self-assessed health questions). Two income questions were asked: monthly income and monthly income in-kind. Total household income was generated by summing both sources of income for all household members. To obtain a crude per capita income measure this total household income could have been divided by the number of people in the household.

However, to proceed in this fashion ignores two issues: different weights for children and adults, and economies of scale [41]. Children typically consume less than adults, and thus counting children as adults may understate the welfare of households with children. For Thailand, empirical studies recommend weighting each child aged under 15 as 0.5 of an adult [42,43]. Also, household members can share some types of goods and services, making them cheaper in households of two or more persons than in one-person households. Economies of scale apply to any household with more than one member, and are incorporated for Thailand by raising weighted household size to the power of 0.75 [42,43]. Thus adult-equivalent monthly household income per household member for households of two or more persons equals total monthly income of all household members divided by (number of adults + 0.5 number of children)^0.75^. For single-person households it equals the monthly income of the single (adult) member.

### Measurement of socioeconomic inequalities in health: Concentration Index

In the recent health economics literature, work on the measurement of inequalities in health using the concentration index has primarily drawn on the literature on income inequality measures [44]. Wagstaff *et al. *provide a critical review and subsequently suggest that only two measures – *the slope and the associated relative index of inequality *and *the concentration curve and the associated concentration index *– meet the minimum criteria for a socioeconomic inequality measure [45]. These criteria are: reflects the socioeconomic dimension of health inequality; reflects the experiences of an entire population; and is sensitive to changes in rank across socioeconomic groups. Their paper also proves the mathematical relationship between the concentration index and the relative index of inequality which is commonly used by epidemiologists.

The concentration index can be written in various ways, one of the most cited being [46]:

C=2nμ∑i=1nhiRi−1
 MathType@MTEF@5@5@+=feaagaart1ev2aaatCvAUfKttLearuWrP9MDH5MBPbIqV92AaeXatLxBI9gBaebbnrfifHhDYfgasaacPC6xNi=xI8qiVKYPFjYdHaVhbbf9v8qqaqFr0xc9vqFj0dXdbba91qpepeI8k8fiI+fsY=rqGqVepae9pg0db9vqaiVgFr0xfr=xfr=xc9adbaqaaeGacaGaaiaabeqaaeqabiWaaaGcbaGaem4qamKaeyypa0tcfa4aaSaaaeaacqaIYaGmaeaacqWGUbGBiiGacqWF8oqBaaGcdaaeWbqaaiabdIgaOnaaBaaaleaacqWGPbqAaeqaaOGaemOuai1aaSbaaSqaaiabdMgaPbqabaGccqGHsislcqaIXaqmaSqaaiabdMgaPjabg2da9iabigdaXaqaaiabd6gaUbqdcqGHris5aaaa@416F@

*h*_*i *_is the health variable of interest for the *ith *person;

*μ *is the mean of *h*;

*R*_*i *_is the *ith*-ranked individual in the socioeconomic distribution from the most disadvantaged (i.e., poorest) to the least disadvantaged (i.e., richest);

*n *is number of persons

As the name implies, the concentration index is a summary measure indicating whether the health (or other) variable of interest is concentrated more at a lower or a higher socioeconomic level. If there is no inequality, it equals 0. If the variable is concentrated at a lower (or higher) socioeconomic level, the concentration index becomes negative (or positive). The larger the absolute value of the concentration index (maximum value = 1.0, minimum value = -1.0), the more pronounced the inequality is. In our data, a concentration index of 0.1 (or -0.1) corresponded to a relative rate (rate ratio) of approximately 2. A relative rate this large, or larger, should be seen as quite substantial for its public health implications [47].

### Decomposing determinants of inequalities in health

In an attempt to explain sources of health inequalities, Wagstaff *et al. *demonstrate that the concentration index of health can be expressed as the sum of contributions of various factors represented by demographic, socioeconomic, and geographic characteristics, together with an unexplained residual component [37]. Based on the linear additive relationship between the health outcome variable *h*_*i*_, the intercept *α*, the relative contributions of *x*_*k *_determinants and the residual error *ε*_*i *_in Equation 2, the concentration index can be rewritten as in Equation 3:

*h*_*i *_= *α *+ ∑_*k*_*β*_*k*_*x*_*ki *_+ *ε*_*i*_

C=∑k(βkx¯kμ)Ck+GCεμ
 MathType@MTEF@5@5@+=feaagaart1ev2aaatCvAUfKttLearuWrP9MDH5MBPbIqV92AaeXatLxBI9gBaebbnrfifHhDYfgasaacPC6xNi=xI8qiVKYPFjYdHaVhbbf9v8qqaqFr0xc9vqFj0dXdbba91qpepeI8k8fiI+fsY=rqGqVepae9pg0db9vqaiVgFr0xfr=xfr=xc9adbaqaaeGacaGaaiaabeqaaeqabiWaaaGcbaGaem4qamKaeyypa0ZaaabeaeaacqGGOaakjuaGdaWcaaqaaGGaciab=j7aInaaBaaabaGaem4AaSgabeaacuWG4baEgaqeamaaBaaabaGaem4AaSgabeaaaeaacqWF8oqBaaaaleaacqWGRbWAaeqaniabggHiLdGccqGGPaqkcqWGdbWqdaWgaaWcbaGaem4AaSgabeaakiabgUcaRKqbaoaalaaabaGaem4raCKaem4qam0aaSbaaeaacqWF1oqzaeqaaaqaaiab=X7aTbaaaaa@457A@

Equation 3 shows that the overall inequality in health outcome has two components, a deterministic or "explained" component and an "unexplained" component; one which cannot be explained by systematic variation in determinants across income groups. In the former component *β*_*k *_is the coefficient from a regression of health outcome on determinant *k*, x¯k
 MathType@MTEF@5@5@+=feaagaart1ev2aaatCvAUfKttLearuWrP9MDH5MBPbIqV92AaeXatLxBI9gBaebbnrfifHhDYfgasaacPC6xNi=xH8viVGI8Gi=hEeeu0xXdbba9frFj0xb9qqpG0dXdb9aspeI8k8fiI+fsY=rqGqVepae9pg0db9vqaiVgFr0xfr=xfr=xc9adbaqaaeGacaGaaiaabeqaaeqabiWaaaGcbaGafmiEaGNbaebadaWgaaWcbaGaem4AaSgabeaaaaa@2EEF@ is the mean of determinant *k*, *μ *is the mean of the health outcome, and *C*_*k *_is the concentration index for determinant *k*. In the latter component, *GC*_*ε *_is the generalised concentration index for the error term.

For the "explained" component, the decomposition framework focuses on two main elements. These are the *impact *each determinant has on health outcomes (βkx¯kμ)
 MathType@MTEF@5@5@+=feaagaart1ev2aaatCvAUfKttLearuWrP9MDH5MBPbIqV92AaeXatLxBI9gBaebbnrfifHhDYfgasaacPC6xNi=xH8viVGI8Gi=hEeeu0xXdbba9frFj0xb9qqpG0dXdb9aspeI8k8fiI+fsY=rqGqVepae9pg0db9vqaiVgFr0xfr=xfr=xc9adbaqaaeGacaGaaiaabeqaaeqabiWaaaGcbaqcfaOaeiikaGYaaSaaaeaaiiGacqWFYoGydaWgaaqaaiabdUgaRbqabaGafmiEaGNbaebadaWgaaqaaiabdUgaRbqabaaabaGae8hVd0gaaiabcMcaPaaa@360D@, and the *degree of unequal distribution *of each determinant across income groups (*C*_*k*_). So, for example, even if the contribution of a determinant is large, if it is equally distributed between rich and poor it will not be a key factor in explaining socioeconomic inequalities in health. Using the decomposition approach, a contribution to an inequality could arise either because a determinant was more prevalent among people of *lower *socioeconomic status and associated with a *higher *probability of reported morbidity or perceived ill health, or because it was more prevalent among people of *higher *socioeconomic status and associated with a *lower *probability of reported morbidity or perceived ill health.

The decomposition method was first introduced to use with a linear, additively separable model [37]. However, because health sector variables are intrinsically non-linear, an appropriate statistical technique for non-linear settings is needed. The two common choices yielding probabilities in the range (0,1) are the *logit *model and the *probit *model, both of which are fitted by maximum likelihood.

hi=αm+∑kβkmxki+ui
 MathType@MTEF@5@5@+=feaagaart1ev2aaatCvAUfKttLearuWrP9MDH5MBPbIqV92AaeXatLxBI9gBaebbnrfifHhDYfgasaacPC6xNi=xI8qiVKYPFjYdHaVhbbf9v8qqaqFr0xc9vqFj0dXdbba91qpepeI8k8fiI+fsY=rqGqVepae9pg0db9vqaiVgFr0xfr=xfr=xc9adbaqaaeGacaGaaiaabeqaaeqabiWaaaGcbaGaemiAaG2aaSbaaSqaaiabdMgaPbqabaGccqGH9aqpiiGacqWFXoqydaahaaWcbeqaaiabd2gaTbaakiabgUcaRmaaqababaGae8NSdi2aa0baaSqaaiabdUgaRbqaaiabd2gaTbaakiabdIha4naaBaaaleaacqWGRbWAcqWGPbqAaeqaaaqaaiabdUgaRbqab0GaeyyeIuoakiabgUcaRiabdwha1naaBaaaleaacqWGPbqAaeqaaaaa@4444@

One possibility when dealing with a discrete change from 0 to 1 is to use *marginal *or *partial effects *(*dh/dx*), which give the change in predicted probability associated with unit change in an explanatory variable. An approximation of the non-linear relationship using marginal effects thus approximately restores the mechanism of the decomposition framework in Equations 2 through 4. So a linear approximation of the non-linear estimations is given by Equation 4, where *u*_*i *_indicates the error generated by the linear approximation used to obtain the marginal effects. Marginal or partial effects have been analysed in the analysis of health sector inequalities in non-linear settings [48,49].

## Results

This section consists of four subsections that follow the steps of the decomposition analysis: i) to obtain the population-weighted proportion and concentration index for each health outcome and each determinant; ii) to obtain marginal effects of the set of determinants for each health outcome variable; iii) to interpret the decomposition results using the 'recently ill' outcome as an example; and iv) to compare the contributions of determinants across the five self-reported morbidity and self-assessed health outcomes.

### i) Poor-rich distribution of health outcomes and their determinants

Table 1 presents the mean and concentration index for each health outcome. The self-reported morbidity variables show that 22 percent of the sample of 37,202 reported having been recently ill (i.e., 'ill or not feeling well' in the last month), while 23 percent reported having suffered from a chronic illness (one which had lasted longer than 3 months) during the past month and 6 percent reported a non-maternity hospital admission during the past 12 months. The self-assessed health variables show that 20 percent of the sample reported their health as being worse or much worse than a year ago, while 13 percent viewed it as being worse or much worse than the health of others of similar age, sex, socioeconomic status and lifestyle (their 'peers').

The crude concentration indices for all five health outcomes were negative, indicating that poorer health was concentrated among the poor. The magnitudes of these inequalities were higher for the self-assessed health outcomes (C = -0.174 for self-assessed health compared to peers and C = -0.139 for self-assessed health compared to a year ago) than for the self-reported morbidity outcomes (C = -0.099, C = -0.085, and C = -0.103 for reported recent, chronic, and hospital inpatient conditions, respectively).

**Figure 1:**
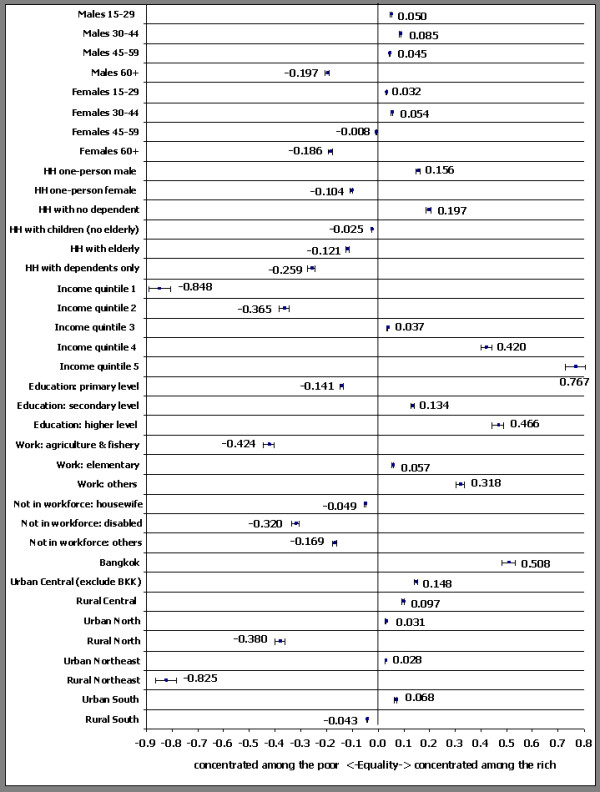
Concentration Indices of determinants (showing 95 percent confidence intervals).

Means for categories of determinants in Table 1 show proportionate distributions of respondents across those categories (i.e., they sum to 1.0). Concentration indices shed light on the poor-rich distributions of determinants. They are presented visually in Figure 1, where the more a concentration index deviates in a positive or negative direction from the vertical line at 0.0 the greater the extent of the inequality in favour of the rich (positive CI) or poor (negative CI).

Persons aged 60 or older were strongly concentrated among the poor (C = -0.197 for males, C = -0.186 for females), while those of prime working age (30–44) tended to be better off (C = 0.085 for males and C = 0.054 for females). It is interesting also to note the gender difference in socioeconomic status of respondents aged 45–59, with males generally better off (C = 0.045) than females (C = -0.008), who may be more financially dependent. As for household type, approximately 28 percent of the sample was drawn from multi-person households consisting of working age persons with no dependent children or elderly, and these people were better off than members of other household types (C = 0.197). Male one-person households also tended to be relatively well off (C = 0.156), but the opposite was true of female one-person households (C = -0.104). Members of multi-person households with dependent children but no elderly (42 percent of the sample), elderly (21 percent of the sample), and no working-age people (5 percent of the sample) were also generally poorer (C = -0.025, C = -0.121, and C = -0.259, respectively).

The income inequality gradient can be clearly observed in Figure 1, concentration indices ranging from C = -0.848 for the lowest quintile to C = 0.767 for the highest one. The socioeconomic gradient in education is also clear. Almost 65 percent of the sample had primary education or less and were poorer (C = -0.141), while those with higher levels of education recorded positive concentration indices (C = 0.134 for the 26 percent who had secondary education and C = 0.466 for the 10 percent with post-secondary education). Around 30 percent of respondents who worked in the agriculture and fishery sector tended to be at the bottom end of the socioeconomic spectrum (C = -0.424), as were the small proportion who were economically inactive because of disability (C = -0.320). The 16 percent not in the workforce for 'Other' reasons that included being unemployed and engaged in full-time education were also relatively poor (C = -0.169), while as was to be expected those employed in less menial occupations (including professionals, technicians, service workers etc.) were socioeconomically well off (C = 0.318).

Concentration indices for geographical areas clearly demonstrate the relatively wealthy and less well-off areas. Fourteen percent of respondents who lived in Bangkok were concentrated at the more advantaged end of the socioeconomic distribution (C = 0.508). The next most advantaged areas were the urban part of the Central region outside Bangkok (C = 0.148) and the rural part of the Central region (C = 0.097), both doubtless benefiting from proximity to the national capital. Rural areas of the other three regions were all socioeconomically disadvantaged. The very high negative concentration index for the rural Northeast (C = -0.825) reflects the high level of poverty in that region noted earlier, but the rural North (C = -0.380) also shows up as strongly disadvantaged. All three of these regions record modest positive urban concentration indices, but that for the urban South (C = 0.068) is the largest of these.

### ii) Marginal effects of determinants

Table 2 shows the marginal effects of each determinant on each of the three self-reported morbidity and two self-assessed health outcome variables obtained by running regressions of determinants on observed probabilities of reporting each outcome based on Equation 4. Marginal effects estimates significant at three levels are highlighted in bold. The reference group was males aged 15–29 years in the top income quintile who had better than secondary education, worked as professionals, technicians or service workers etc., lived in a multi-person household without dependents, and resided in the urban part of Central Thailand outside of Bangkok.

**Table 2:** Probability of determinants on reporting health outcome variables

**Determinants**	**Recent illness**	**Chronic illness**	**Hospital admission**	**Health compared to a year ago**	**Health compared to peers**
***Demographic characteristics***					
***Age-sex***					
Males aged 30–44	0.058***	0.108***	0.012	0.135***	0.080***
Males aged 45–59	0.146***	0.230***	0.019*	0.270***	0.112***
Males aged 60+	0.302***	0.470***	0.083***	0.503***	0.243***
Females aged 15–29	0.062***	0.064**	-0.014	0.059**	0.024
Females aged 30–44	0.161***	0.212***	0.001	0.179***	0.114***
Females aged 45–59	0.245***	0.372***	0.026*	0.325***	0.176***
Females aged 60+	0.403***	0.554***	0.078***	0.559***	0.289***
***Household type***					
One-person male household	0.067**	0.033	-0.005	0.015	0.018
One-person female household	0.087***	0.017	-0.002	0.007	0.006
Household with children (no elderly)	-0.012	0.009	-0.008	-0.001	-0.005
Household with elderly	-0.037**	-0.008	-0.011*	-0.042***	-0.028**
Household with dependents only	-0.020	-0.009	-0.017*	-0.039**	-0.024*
***Socioeconomic characteristics***					
***Income***					
Income quintile 1 – lowest 20%	0.031*	-0.010	0.004	0.054***	0.048***
Income quintile 2 – lower 20%	0.032*	-0.020	0.006	0.031*	0.020
Income quintile 3 – middle 20%	0.001	-0.016	0.002	0.015	0.014
Income quintile 4 – higher 20%	0.018	-0.020	-0.001	0.018	0.007
***Education***					
Education: primary level	0.052***	0.069***	0.001	0.072***	0.059***
Education: secondary level	0.015	0.017	-0.007	0.000	0.013
***Work status***					
Work: agriculture and fishery	-0.009	-0.021*	-0.006	-0.004	-0.016*
Work: elementary occupation	-0.029*	-0.017	0.001	0.002	0.009
Not in workforce: housewife	-0.033*	-0.003	0.002	-0.009	0.000
Not in workforce: disabled	0.250***	0.451***	0.154***	0.292***	0.532***
Not in workforce: others	-0.010	0.012	0.017**	0.017	0.015
***Geographic characteristics***					
Bangkok	0.016	0.019	-0.027***	-0.010	0.013
Rural Central	0.041*	0.042**	-0.005	0.011	0.015
Urban North	0.112***	0.078***	0.008	0.006	0.024*
Rural North	0.142***	0.108***	0.011	0.006	0.041**
Urban Northeast	0.004	0.031*	0.007	0.029	0.048***
Rural Northeast	0.039*	0.042**	0.001	0.035*	0.050***
Urban South	0.063**	0.075***	-0.003	0.058**	0.046**
Rural South	0.079***	0.039*	-0.004	0.055**	0.041**

Note: The marginal effects demonstrate associations between determinants and health outcomes. Those with positive signs indicate positive associations with the probability of reporting a health outcome, while those with negative signs indicate negative associations. In addition, the larger the absolute value of a marginal effect, more substantial is the association. Statistically significant estimates of marginal effects are highlighted (*p < 0.05, **p < 0.01, ***p < 0.001). Reference groups were males aged 15–29; household with no dependent; income quintile 5; work status: others including professionals, technicians, or service workers; and urban Central excluding Bangkok. Elementary occupation includes the likes of street vendors, domestics, and non-agricultural labourers.

The marginal effects demonstrate associations between determinants and health outcomes. Those with positive signs indicate positive associations with the probability of reporting a health outcome, while those with negative signs indicate negative associations. In addition, the larger the absolute value of a marginal effect, more substantial is the association. Increasing age was significantly associated with increased probabilities of reporting morbidity and assessing one's health adversely, effects being consistently largest for respondents aged 60 or older. Age and sex had large effects on morbidity (reported chronic illness and illness requiring hospitalization) as well as self-assessed health compared to a year ago. This is intuitively sound because the biological process of aging is known to be associated with both types of morbidity and with deterioration in self-assessed health over time. As for household type, being in a one-person household was particularly associated with higher probabilities of reporting recent illness, while living in a household including at least one elderly dependent was associated with slightly lower probabilities of reporting all outcomes except chronic illness. The interpretation to be placed on this small effect is unclear, but it might reflect (i) a tendency for co-residence with elderly and exposure to their health concerns to cause one to take a more optimistic view of one's own health and/or (ii) a tendency in households where elderly couples co-reside for healthier partners to have selectively responded to self-reported morbidity and self-assessed health items.

Probabilities of reporting recent illness, chronic illness, hospital admission, or of adversely assessing one's health compared to a year ago or compared to peers could in some cases be explained by socioeconomic determinants. The lowest two income quintiles had significant positive associations with probabilities of reporting recent illness, a deterioration in health over the past year, and inferior health compared to peers. A low level of education similarly had strong, positive associations with reporting all five health outcomes except hospitalization. Despite having lower socioeconomic status, respondents employed in 'agriculture and fishery' reported significantly less chronic illness and were a little less likely to rate their health poorer than that of peers. These small effects could point to these occupations requiring a basic level of physical wellbeing and to chronic health problems being likely to exclude people from them, but results for this determinant are considered in more detail in the Discussion section below. Those in 'elementary' occupations and housewives were significantly less likely to have reported being recently ill, perhaps suggesting higher tolerance of minor ailments among groups vital to household functioning. Unsurprisingly, being out of the workforce due to disability substantially associated with all five health outcome variables, but especially with reporting chronic illness and worse health than was typical for one's cohort.

Geographically, residence anywhere outside of Bangkok and other urban areas of the Central region was associated with significantly more reporting of chronic illness, while residence in Bangkok was associated with a significantly lower level of hospitalization during the preceding year. Recent illness was also more often reported outside Bangkok and urban Central Thailand, except in the urban Northeast. Rating one's health inferior to that of peers was significantly more common in all geographic areas outside the Central region, but assessing it as having deteriorated over the previous 12 months was common only in the South and the rural Northeast. Whether this finding reflects recent political unrest in the South and the relatively high poverty level in the rural Northeast are moot points.

### iii) Interpretation of decomposition results: the case of recent illness

In this subsection one of the five outcome variables studied, recent illness, is taken as an example to illustrate the decomposition of a concentration index into its determinants. The following subsection then compares decomposition results across all five outcome variables. Computed using Equation 3, column 5 of Table 3 is a by-product of how the marginal effects, means and concentration indices of determinants translate into absolute contributions to the total observed socioeconomic inequality in health. In this illustrative example, the crude concentration index for recent illness was -0.099, indicating that claims to have been 'ill or not feeling well' during the preceding month were concentrated amongst the poor. The absolute contribution of each determinant (column 5) is obtained by multiplying its marginal effect by its mean and concentration index, then dividing by the mean of the health outcome (the proportion reporting that outcome). Positive (negative) contributions of determinants can be interpreted as indicating that the total health inequality would, *ceteris paribus*, be lower (higher) if that determinant had no impact on the health outcome (instead of that reflected in marginal effects, column 2) or was equally distributed across the socioeconomic spectrum (instead of concentrated, as mirrored in the concentration indices of determinants, column 4).

**Table 3:** Decomposition results: illustrative example for reports of recent illness

(Col 1)	(Col 2)	(Col 3)	(Col 4)	(Col 5)	(Col 6)	(Col 7)
**Determinant**	**Marginal effect**	**Weighted proportion**	**Concentration Index (***C*_*k*_**)**	**Deterministic contribution **C _*recentillness *_**-0.099**	**Unadjusted percentage contribution**	**Adjusted percentage contribution**
***Demographic characteristics***						
***Age-sex***						
Males aged 30–44	0.058***	0.144	0.085	0.003	-2.2	
Males aged 45–59	0.146***	0.103	0.045	0.003	-2.1	
Males aged 60+	0.302***	0.061	-0.197	-0.016	11.3	(8.6%)
Females aged 15–29	0.062***	0.170	0.032	0.002	-1.1	
Females aged 30–44	0.161***	0.176	0.054	0.007	-4.7	
Females aged 45–59	0.245***	0.123	-0.008	-0.001	0.7	(0.5%)
Females aged 60+	0.403***	0.079	-0.186	-0.026	18.3	(13.9%)
			***Subtotal***	**-0.029**	**20.2%**	**(23.0%)**
***Household type***						
One-person male household	0.067**	0.023	0.156	0.001	-0.7	
One-person female household	0.087***	0.022	-0.104	-0.001	0.6	(0.5%)
Household with children (no elderly)	-0.012	0.279	-0.025	0.001	-0.4	
Household with elderly	-0.037**	0.209	-0.121	0.004	-2.9	
Household with dependents only	-0.020	0.047	-0.259	0.001	-0.8	
			***Subtotal***	**0.006**	**-4.2%**	**(0.5%)**
***Socioeconomic characteristics***						
***Income***						
Income quintile 1 – lowest 20%	0.031*	0.236	-0.848	-0.028	19.4	(14.7%)
Income quintile 2 – lower 20%	0.032*	0.211	-0.365	-0.011	7.5	(5.7%)
Income quintile 3 – middle 20%	0.001	0.181	0.037	0.000	0.0	
Income quintile 4 – higher 20%	0.018	0.188	0.420	0.006	-4.3	
***Education***						
Education: primary level	0.052***	0.641	-0.141	-0.021	14.6	(11.1%)
Education: secondary level	0.015	0.258	0.134	0.002	-1.6	
***Work status***						
Work: agriculture and fishery	-0.009	0.295	-0.424	0.005	-3.7	
Work: elementary occupation	-0.029*	0.090	0.057	-0.001	0.4	(0.3%)
Not in workforce: housewife	-0.033*	0.081	-0.049	0.001	-0.4	
Not in workforce: disabled	0.250***	0.015	-0.320	-0.006	3.8	(2.9%)
Not in workforce: others	-0.010	0.164	-0.169	0.001	-0.8	
			***Subtotal***	**-0.050**	**34.9%**	**(34.8%)**
***Geographic characteristics***						
Bangkok	0.016	0.139	0.508	0.005	-3.5	
Rural Central	0.041*	0.142	0.097	0.002	-1.7	
Urban North	0.112***	0.041	0.031	0.001	-0.4	
Rural North	0.142***	0.154	-0.380	-0.037	25.7	(19.5%)
Urban Northeast	0.004	0.055	0.028	0.000	0.0	
Rural Northeast	0.039*	0.286	-0.825	-0.041	28.4	(21.6%)
Urban South	0.063**	0.024	0.068	0.000	-0.3	
Rural South	0.079***	0.086	-0.043	-0.001	0.9	(0.7%)
			***Subtotal***	**-0.071**	**49.1%**	**(41.8%)**

		***Residual (unexplained)***		***0.045***		

Note: Reference groups were males aged 15–29; household with no dependent; income quintile 5; work status: others including professionals, technicians, or service workers; and urban Central excluding Bangkok. Readers could calculate the contributions of determinants for other health outcomes according to Equation 3 by using means and concentration indices of determinants provided in Table 1 and marginal effects provided in Table 2.

To obtain the corresponding unadjusted percentage contributions (column 6), each absolute contribution is divided by the total explained portion of the concentration index (i.e., in this example, -0.029 +0.006 -0.050 -0.071 = -0.144; or the overall concentration index (-0.099) minus the residual 0.045). These unadjusted percentages are a mixture of positives and negatives, in which the positives need both to offset the negatives *and then *sum to 100 (i.e., the positives sum to in excess of 100 by an amount equal to the absolute value of the sum of the negative percentages). To quote positive unadjusted percentages for individual determinants or groups of determinants as indicators of their contribution to the explained portion of the concentration index therefore exaggerates their importance. Adjusted percentages reduce positive unadjusted percentages to levels that sum to 100 on the assumption that each positive unadjusted percentage contributes *pro rata *to the offsetting of negative unadjusted percentages.

In the example in Table 3, where recent illness is concentrated among the poor (negative concentration index), contributions of individual determinants to the overall inequality can be interpreted as follows. Females aged 60 years and older had an above average probability of reporting recent illness (positive marginal effect, column 2), were disproportionately concentrated in lower income groups (negative concentration index, column 4), and thus contributed -0.026, or 13.9 percent, to the total observed inequality in recent illness (columns 5 and 7). Since this contribution has the same sign as the overall concentration index, which indicates that recent illness was concentrated amongst the poor, it is one that signifies that elderly females were a major reservoir of poor people who reported recent illness. With this approach it is possible to compare contributions across categories of a characteristic, such as among the different age-sex groups. For example, the 13.9 percent contribution of elderly females to reported recent illness was higher than the 8.6 percent contribution of their elderly male counterparts.

The interpretation of a contribution with opposite sign to that on the concentration index can be illustrated as follows. Females aged 30–44 years had a higher probability of reporting a recent illness than males aged 15–29 (the reference group) – a positive marginal effect in column 2. But because they were disproportionately in the higher income group (positive concentration index in column 4), their contribution of 0.007 in column 5 was in the opposite direction to the overall inequality observed.

In looking at socioeconomic determinants, the combination of a positive association between being in the lowest income quintile and reported recent illness (positive marginal effect in column 2) and especially this lowest quintile being an ultra-poor group (large negative concentration index in column 4) results in being in the lowest income quintile contributing -0.028, or 14.7 percent (columns 5 and 7), to the total inequality in reported recent illness. With a positive association between low education and reported recent illness (positive marginal effect in column 2) and those with low education being concentrated at lower income levels (negative concentration index in column 4), low education also contributed -0.021, or 11.1 percent, to the total inequality.

Concerning geographical contributions, residence in the rural North and rural Northeast was positively associated with reporting recent illness (positive marginal effects in column 2), and since these rural dwellers were disproportionately poor (large negative concentration indices in column 4) they contributed -0.037 (rural North) and -0.041 (rural Northeast), or a combined total of 41.1 percent, to the total observed inequality in recent illness.

### iv) Comparing and contrasting decomposition results for self-reported morbidity and self-assessed health outcomes

Table 4 compares and contrasts decomposition results for the three self-reported morbidity and two self-assessed health outcomes. It captures the equivalents of columns 5 and 7 of Table 3 for all five decomposition analyses. The first row presents crude concentration indices and the second shows age-sex-adjusted concentration indices calculated by deducting the contributions of age-sex determinants from crude indices. The age-sex structure of samples is known to be a confounder in the study of socioeconomic health inequalities [46]. Retired elderly, for example, tend to have lower incomes, but because they are older tend also to be sicker. The advantage of presenting full decomposition results is that it provides a convenient way to subtract the contribution of age and sex from the crude concentration index.

**Table 4:** Decomposition results: contributions of determinants to Concentration Indices (absolute value and adjusted percentage of total explanatory variables)

	**Recent illness**	**Chronic illness**	**Hospital admission**	**Health compared to a year ago**	**Health compared to peers**
**Concentration Index**	**-0.099**	**-0.085**	**-0.103**	**-0.139**	**-0.174**
**Age-sex adjusted CI**	**-0.070**	**-0.043**	**-0.068**	**-0.090**	**-0.138**

***Demographic characteristics***					
***Age-sex***					
Males aged 30–44	0.003	0.006	0.002	0.008	0.008
Males aged 45–59	0.003	0.005	0.002	0.006	0.004
Males aged 60+	-0.016 (8.6%)	-0.025 (13.8%)	-0.017 (12.4%)	-0.031 (13.3%)	-0.022 (7.1%)
Females aged 15–29	0.002	0.002	-0.001 (1.0%)	0.002	0.001
Females aged 30–44	0.007	0.009	0.000	0.008	0.008
Females aged 45–59	-0.001 (0.5%)	-0.002	0.000 (0.3%)	-0.002 (0.7%)	-0.001 (0.4%)
Females aged 60+	-0.026 (13.9%)	-0.036 (19.8%)	-0.020 (14.2%)	-0.041 (17.9%)	-0.033 (10.3%)
	**-0.029 (23.0%)**	**-0.042 (33.7%)**	**-0.034 (27.8%)**	**-0.049 (31.9%)**	**-0.036 (17.9%)**
***Household type***					
One-person male household	0.001	0.001	-0.000 (0.2%)	0.000	0.000
One-person female household	-0.001 (0.5%)	0.000 (0.1%)	0.000	0.000 (0%)	0.000 (0%)
HH with children (no elderly)	0.001	0.000 (0.2%)	0.001	0.000	0.000
HH with elderly	0.004	0.001	0.005	0.005	0.005
HH with dependents only	0.001	0.000	0.004	0.002	0.002
	**0.006 (0.5%)**	**0.001 (0.3%)**	**0.010 (0.2%)**	**0.008 (0%)**	**0.008 (0%)**
***Socioeconomic characteristics***					
***Income***					
Income quintile 1	-0.028 (14.7%)	0.009	-0.015 (10.9%)	-0.054 (23.5%)	-0.074 (23.4%)
Income quintile 2	-0.011 (5.7%)	0.007	-0.008 (5.6%)	-0.012 (5.3%)	-0.012 (3.7%)
Income quintile 3	0.000	-0.000 (0.3%)	0.000	0.000	0.001
Income quintile 4	0.006	-0.007 (3.9%)	-0.001 (0.7%)	0.007	0.004
***Education***					
Education: primary level	-0.021 (11.1%)	-0.028 (15.3%)	-0.002 (1.4%)	-0.033 (14.1%)	-0.041 (12.9%)
Education: secondary level	0.002	0.003	-0.004 (3.1%)	0.000 (0%)	0.003
***Work status***					
Work: agriculture and fishery	0.005	0.012	0.012	0.002	0.015
Work: elementary occupation	-0.001 (0.3%)	-0.000 (0.2%)	0.000	0.000	0.000
Not in workforce: housewife	0.001	0.000	-0.000 (0.1%)	0.000	0.000 (0%)
Not in workforce: disabled	-0.006 (2.9%)	-0.010 (5.4%)	-0.013 (9.3%)	-0.007 (3.1%)	-0.020 (6.4%)
Not in workforce: others	0.001	-0.001 (0.8%)	-0.008 (5.9%)	-0.002 (1.0%)	-0.003 (1.0%)
	**-0.050 (34.8%)**	**-0.017 (25.9%)**	**-0.038 (37.0%)**	**-0.098 (47.1%)**	**-0.125 (47.4%)**
***Geographic characteristics***					
Bangkok	0.005	0.006	-0.032 (23.0%)	-0.003 (1.5%)	0.007
Rural Central	0.002	0.003	-0.001 (0.8%)	0.001	0.002
Urban North	0.001	0.000	0.000	0.000	0.000
Rural North	-0.037 (19.5%)	-0.028 (15.5%)	-0.011 (7.7%)	-0.002 (0.8%)	-0.018 (5.9%)
Urban Northeast	0.000	0.000	0.000	0.000	0.001
Rural Northeast	-0.041 (21.6%)	-0.044 (24.3%)	-0.005 (3.4%)	-0.042 (18.3%)	-0.090 (28.5%)
Urban South	0.000	0.001	0.000 (0.1%)	0.000	0.001
Rural South	-0.001 (0.7%)	-0.001 (0.4%)	0.000	-0.001 (0.4%)	-0.001 (0.4%)
	**-0.071 (41.8%)**	**-0.063 (40.2%)**	**-0.048 (35.0%)**	**-0.047 (21.0%)**	**-0.099 (34.7%)**

***Residuals (unexplained)***	***0.045***	***0.036***	***0.009***	***0.047***	***0.078***

Note: Reference groups were males aged 15–29; household with no dependent; income quintile 5; work status: others including professionals, technicians, or service workers; and urban Central excluding Bangkok.

Across the five health outcomes, age and sex played particularly significant explanatory roles in inequality by income in reported chronic illness (33.7 percent), non-maternity hospitalizations (27.8 percent) and deterioration in health compared to a year ago (31.9 percent). Females aged 60 or older made the strongest contribution of any individual demographic determinant across all five health outcomes (Table 4), and except for hospital admissions marginal effects for females were generally stronger than those for males in all age groups (Table 2). This supports literature suggesting that females have a greater tendency to report morbidity [50].

Notably, socioeconomic determinants explained one-third or more of the total inequalities observed, except in the case of chronic illness (Table 4). They were particularly important in respect of poorer self-perceived health compared to a year ago and compared to peers. Being in the lowest income quintile and having no more than primary education contributed 14.7 and 11.1 percent, respectively, to inequalities unfavourable to the poor in reported recent illness. But the effects of being in the bottom income quintile were much stronger upon inequalities in the two measures of adverse self-assessed health than upon those in the three self-reported measures of morbidity. Being in this quintile contributed 23.5 and 23.4 percent to the tendencies of the poor to more often report their health to be worse or much worse compared to a year ago and compared to their peers. On the other hand it contributed nothing to their tendency to more often report chronic illness; low education was the major socioeconomic determinant here, and was also prominent in accounting for all other outcomes except hospitalization. Those who worked in the agricultural and fishery sector were non-contributors to overall inequality favouring the poor on all five health outcomes. This is a surprising finding given that such people are typically of lower socioeconomic status and exposed to certain health hazards in the course of their employment. One possibility is that this determinant overlaps with, and is suppressed by, those measuring low income and education. It is given credence by the finding that when models were refitted with income and education determinants excluded, contributions of the agricultural and fishery sector increased considerably and had the same sign as the overall concentration indices. Beyond this, it is possible that the physical demands of agriculture and fishery work require a relatively high level of wellbeing and fitness, engender a rather stoic attitude to ill-health and cause people to cease employment and join a 'Not in workforce' category should chronic or serious medical conditions afflict them. Restricted access to health care could also be a factor, limiting awareness of chronic conditions and lowering the hospital admission rate. The emphasis on primary health care in rural areas associated with the Universal Coverage Scheme may also be reflected in the lack of association of both this determinant and low education with greater hospitalization among the poor. The only work status determinant to contribute consistently to health outcomes unfavourable to the poor was disability, its strongest effects being on hospital admission (9.3 percent), chronic illness (5.4 percent) and assessing one's health to be inferior to that of peers (6.4 percent).

Geographic determinants yielded some interesting results. Holding everything else constant, living in the rural Northeast and the rural North were important contributors to inequality in reported recent and chronic illness (21.6 percent and 24.3 percent, respectively, in the Northeast; 19.5 percent and 15.5 percent in the North). In the case of hospitalization there were also much smaller contributions to inequality favouring the poor from residence in these two areas, but the major geographic determinant was residence in Bangkok (23.0 percent), clearly reflecting the importance to hospitalizations of the poor of proximity to major hospitals. The extent to which this may point to unnecessary, supplier-induced admissions in Bangkok or to sub-optimal access to hospitals elsewhere in the country is a moot point.

## Discussion

This paper has sought to help fill the gap in information about socioeconomic inequalities in self-reported morbidity and self-assessed health in developing countries, and also to decompose inequalities to reveal their determinants. In the case of Thailand, each of five adverse health outcomes was concentrated among the poor.

Comparing across health outcome variables, older age, particularly in conjunction with being female, was the main contributor to inequality in reported chronic illness and perceived deterioration in health over the previous year. Beyond that, low socioeconomic status as indexed by low income and low education, respectively, was a major contributor to the inequality in reports of recent and chronic illness. Being in the lowest income quintile and having primary or less education also contributed strongly to the wider perceptions among the poor that their health was inferior to that of peers and inferior to what it had been a year previously. Geographically, residing in the rural Northeast and the rural North were main contributors to inequalities in reported recent and chronic illness, and perhaps reflecting a much higher level of poverty than in other parts of the country residence in the rural Northeast was also strongly linked to the poor more often assessing their health to have deteriorated recently and to be worse than that of peers. If there were no systematic regional disparities, in other words, the overall inequalities observed would be lower.

It is instructive to note one or two broad differences between models for the five outcome variables in Table 4. First, socioeconomic determinants are decidedly less important in producing inequality in chronic illness than in producing inequality in the other four health outcomes, and to the extent that they *are *relevant it is low education, not low income, that stands out. Chronic illness is concentrated among the poor primarily because of associations with old age, with lack of education (which probably inhibits preventive behaviour), and with living in the rural North and Northeast, where health services are poorest. Second, relative poverty is a determinant of the tendency to be admitted to hospital for non-maternity reasons. There is naturally an association with old age, but the poverty determinants (low income, disability and probable unemployment specified as 'Not in workforce: others') produce the overall socioeconomic effect. Geographically, residence in Bangkok and thus proximity to hospital services, is an important determinant of unequal use of health services. The Thai Universal Coverage Scheme has deliberately set out to promote primary health care facilities as first ports of call for the poor, thereby discouraging unnecessary patronage of the hospital system. Finally, the finding that the rural Northeast alone stands out as a geographic determinant of the poor being more likely to believe that their health is deteriorating and is inferior to that of peers is notable. It suggests an acute awareness among residents of this region of the disadvantage and poverty with which they contend compared to other parts of the country, and perhaps even suggests a somewhat fatalistic outlook on life.

Despite rising levels of development, self-reported and self-assessed health questions in health surveys in developing countries have not been fully utilized. In the case of Thailand, the national Health and Welfare Surveys (HWSs) have included self-reported morbidity questions for decades, but self-assessed health questions were only introduced in 2003. Results from the present analysis of 2003 data have shown that socioeconomic inequalities unfavourable to the poor arise with respect to both self-reported morbidity and self-assessed health. Earlier studies based on the HWSs of 1986, 1991 and 1996 also showed that illnesses of all types, recent, chronic and those requiring hospitalization, were concentrated among the poor [51,52]. The addition of self-assessed health questions to the HWS from 2003 has augmented significantly its potential for facilitating studies of health inequity and future trends therein [53,54].

There are some issues to be considered concerning the nature of the data used here. While household health surveys are quite common in developing countries, the reliability of data from them for studies of socioeconomic inequalities in health has at times been questioned. Baker and van der Gaag, for example, found that in Ghana, Jamaica, Peru and Bolivia (but not in Côte d'Ivoire), the better off were more likely than the poor to report themselves ill [10]. These unexpected results were based on responses to a question inquiring whether household survey respondents had been ill in the past month [55,56]. They raise the issue of perception bias; the notion that people at different socioeconomic levels may have different perceptions of what constitutes being 'ill'. Such differences can reflect variations in cultural norms, knowledge and beliefs that among other things create different thresholds of tolerance of debility, or different degrees of stoicism in the face of adversity. They include variations in what is normative by way of life's daily rigours, in what degree of incapacity justifies withdrawal from normal daily activity, and in knowledge necessary to recognise certain adverse health conditions and appreciate the capacity of medical services to treat them [57]. Results of studies of health inequalities can be biased by inclinations in certain subgroups of respondents to over- or under-report health problems. In the present study, the finding that employment in the agriculture and fishery sector did not contribute to inequalities disadvantaging the poor on any of the five health outcomes examined might, given such people's low socioeconomic status, reflect perception bias. Then again it could be a product of statistical suppression of this determinant due to substantial overlap with low income and low education. It is certainly possible that a socioeconomically poorer group might, for example, under-report chronic illness through lack of awareness stemming from poorer access to healthcare [57,58].

There are some limitations to this study. Caution is required reaching causal conclusions from it. Its design is cross-sectional, and thus data on outcomes and determinants were collected simultaneously. Future studies could usefully make use of cohort or longitudinal data to better understand changes in socioeconomic status and their link to changes in self-reported morbidity and self-assessed health. The decomposition approach is deterministic, and only includes measured explanatory variables. There are clearly other cultural, community, or health system determinants besides the demographic, socioeconomic and geographic characteristics examined here which contribute to inequalities [56]. Lastly, this study has used binary outcome variables. Further studies using continuous outcome variables from linear regression or other types of generated data may provide more information.

## Conclusion

Using decomposition analysis this paper shows that certain demographic, socioeconomic and geographic characteristics were particularly associated with poor-rich differences in reported health status in Thailand in 2003. Being older, particularly in conjunction with being female, was the main contributor to inequality in self-reported morbidity, especially in relation to reported chronic illness and illness requiring hospital admission. In addition, having low socioeconomic status as reflected in low education and being in the bottom income quintile contributed over one-third of the overall inequalities in perceiving one's health status to be inferior to that of peers and inferior to what it was a year ago. Geographically, residing in the rural North and the rural Northeast contributed around forty percent to inequalities in reported recent and chronic illness. But concerning the poor's tendency to more often assess their health as worse than that of peers it is residence in the rural Northeast alone that stands out. The relative poverty of this area may lead respondents to compare themselves to peers not only locally but nationally, and manifest an acute sense of disadvantage compared to the rest of Thailand.

The decomposition results are consistent with findings of a recent qualitative study of equity in the health system in Thailand. This found socioeconomic and geographic inequalities in health to be of great concern, and to have major implications for health care utilization [59]. Self-reported morbidity and self-assessed health are important concepts in assessing the demand for health services. Some self-reported chronic conditions, for example, generally follow from diagnosis by a health professional, and thus those with less access to and/or less utilization of the health system may, through lack of awareness, be less likely to report such conditions.

Thailand is an interesting case among developing countries as it has attempted to address concern over inequalities in health-related outcomes, and in particular in access to and use of health services, by introducing a universal coverage health insurance policy. In order to advance equity in access to healthcare, studies need to continue to monitor differential health outcomes and the differential use of health services as the universal coverage era unfolds [60].

## Competing interests

The author(s) declare that they have no competing interests.

## Authors' contributions

VY designed the study, analysed data, and drafted the manuscript. LL was involved in the conceptualisation of the manuscript, interpretation of data and provided statistical advice throughout the study. GC contributed to the interpretation of data and provided detailed commentary and editorial guidance, which substantially improved various versions of the manuscript. AS contributed to technical elucidation of the concentration index and the decomposition model. ACS played an advisory role in all aspects of the study. All authors read and approved the final version of the manuscript.
